# Rapid quantification of stroke volume using magnetohydrodynamic voltages in 3T MRI: a feasibility study

**DOI:** 10.1186/1532-429X-17-S1-P32

**Published:** 2015-02-03

**Authors:** Stan T Gregory, Ehud J Schmidt, Shelley H Zhang, Raymond Y Kwong, William G Stevenson, John Oshinski, Zion T Tse

**Affiliations:** 1 University of Georgia, Athens, GA, USA; 2 Brigham and Women's Hospital, Boston, MA, USA; 3 Emory University Hospital, Atlanta, GA, USA

## Background

The Electrocardiogram (ECG) is a standard clinical tool for cardiac physiological monitoring, required for cardiac synchronization during cardiac MRI, despite signal artifacts resulting from Magnetohydrodynamic voltages (VMHD). VMHD becomes significant during systole when rapidly ejected blood from the left ventricle into the aortic arch interacts with the strong magnetic field (B_0_) of the MRI [1]. Due to this relationship, we hypothesized that blood flow as a function of time in the cardiac cycle and left ventricular Stroke Volume (SV) could be derived using VMHD extracted from intra-MRI ECG. This method would allow for real-time beat-to-beat SV estimation during clinical MR scanning and cardiac MRI stress testing. This non-invasive real-time physiological measure of patient condition can be provided with the described software processing during conventional cardiac MRI routines, and potentially replace Invasive Blood Pressure during complex interventional procedures.

## Methods

**Figure 1:**
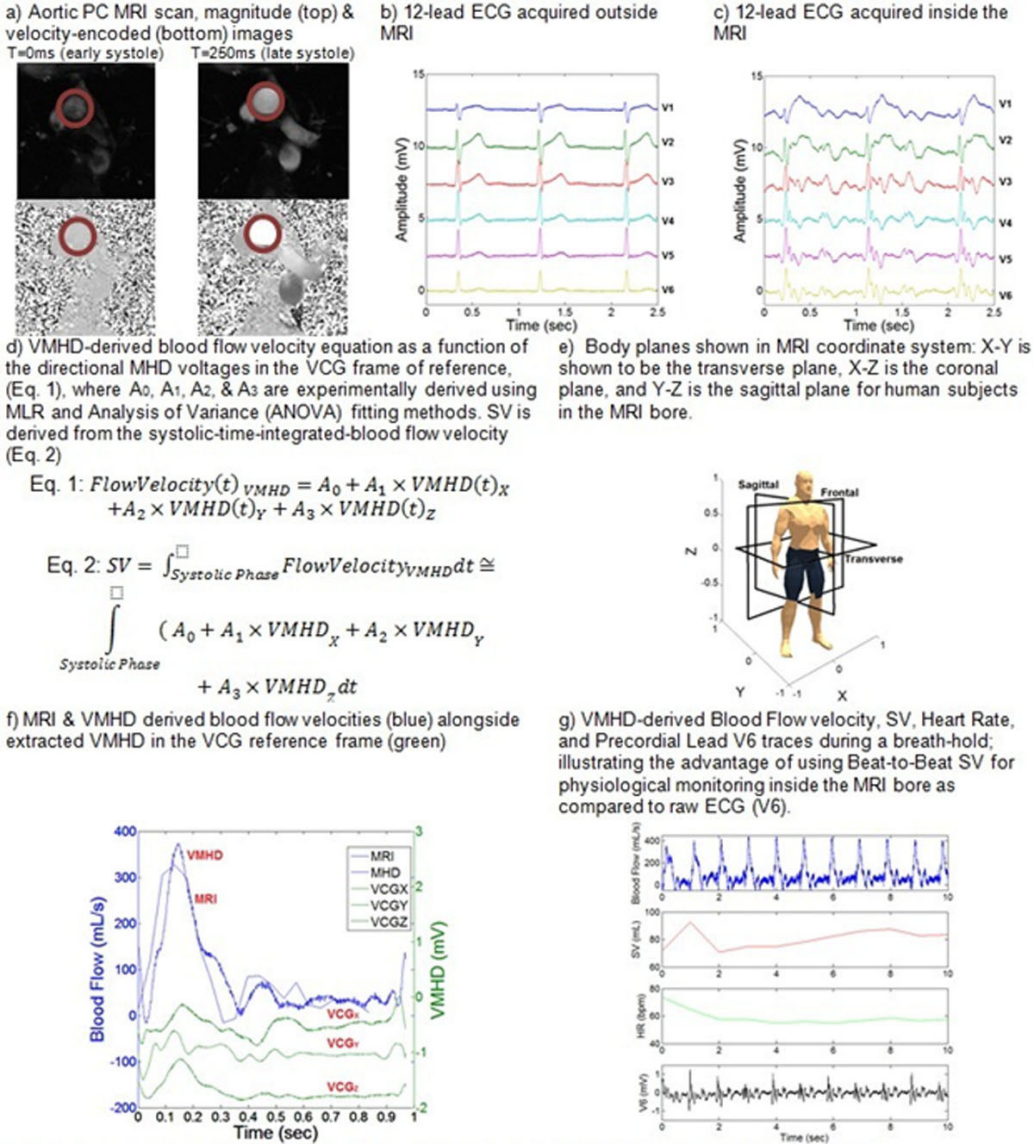
Stroke volume estimation using VMHD and VENC PC based methodology in human subject#1

Velocity-Encoded (VENC) Phase Contrast Cine MRI slices were obtained in three healthy volunteer subjects (n=3) along the aortic arch (Fig. 1a) to quantify the volume of blood flow and SV using a Siemens Skyra 3T MRI scanner with the following scan parameters: VENC: 150 cm/s; TR: 44.08 ms; TE: 3.28ms; and Flip Angle: 20^o^. A GE digital-IT 12-Lead ECG recording system modified to be MRI-compatible [2,3] was used to record 12-lead ECG traces from the subjects at 3T (Fig. 1b-c). VMHD voltages at each electrode were extracted through the subtraction of ECGs obtained with the subjects outside and inside the MRI [2]. The 12-lead VMHD traces were converted into the Vectorcardiograms (VCG) frame of reference using an inverse Dower transform [4]. For each subject, a multiple linear regression (MLR) (Fig. 1d) was used to correlate VMHD in the VCG reference frame (Fig. 1e) to blood flow volume as a function of time obtained using Cine PC MRI scans over 33 cardiac cycles (Fig. 1f-g). VMHD-derived blood flow velocity was then time-integrated over the systolic cardiac phase to estimate SV, and compared to the "gold-standard" derived from PC MRI.

## Results

After MLR was performed for each subject (Table 1), aortic blood flow as a function of time was computed from the VMHD(t) and compared through VENC PC MRI to evaluate fit, with correlation determined through a Spearman's Ranked Coefficient, found to be > 0.84 (Fig. 1f). VMHD-based SV was determined with a < 3.6% error as compared to PC MRI in all three subjects. Fig. 1g shows the real-time beat-to-beat blood flow velocity and SV derived from VMHD, as well as the associated Heart Rate (HR) and raw ECG channel (Precordial Lead V6).

**Table 1:** Multiple Linear Regression for Blood Flow and Stroke Volume Estimation using VMHD

Subject	A0	A1	A2	A3	Cross-Correlation	VMHD SV	PC SV	SV Error
1	63.3	45.7	-195.1	-247.4	0.94	73.7 mL	75.7 mL	2.62 %

2	70.5	152.4	-331.3	-316.2	0.95	78.5 mL	78.1 mL	0.59 %

3	106.4	248.1	-285.4	-274.0	0.84	55.1 mL	53.2 mL	3.56 %

## Conclusions

Relatively accurate beat-to-beat stroke volume and blood flow velocity estimates can be obtained from MHD voltages extracted from 12-lead ECG, providing a means for enhanced patient monitoring inside the MRI bore. A relatively short Phase-Contrast Cine measurement is required to provide the required patient-specific parameters.

## Funding

NIH U41-RR019703, NIH R03 EB013873-01A1, SBIR-1 R43 HL110427-01.
